# Effectiveness of Graded Weight-Bearing Exercises on Pain, Function, Proprioception, and Muscle Strength in Individuals with Knee Osteoarthritis: A Randomized Controlled Trial

**DOI:** 10.3390/jcm14217685

**Published:** 2025-10-29

**Authors:** Ammar Fadil, Qassim Ibrahim Muaidi, Mohamed Salaheldien Alayat, Moayad S. Subahi, Roaa A. Sroge, Abdulaziz Awali, Mansour Abdullah Alshehri

**Affiliations:** 1 Department of Medical Rehabilitation Sciences, Faculty of Applied Medical Sciences, Umm Al-Qura University, Mecca 24382, Saudi Arabia; asfadil@uqu.edu.sa (A.F.); msayiat@uqu.edu.sa (M.S.A.); mssubahi@uqu.edu.sa (M.S.S.); rasroge@uqu.edu.sa (R.A.S.); amawali@uqu.edu.sa (A.A.); 2 Department of Physical Therapy, College of Applied Medical Sciences, Imam Abdulrahman Bin Faisal University, Dammam 31441, Saudi Arabia; qmuaidi@iau.edu.sa

**Keywords:** knee osteoarthritis, knee pain, weight-bearing exercise, closed kinetic chain exercise, physiotherapy

## Abstract

**Background/Objectives:** Knee osteoarthritis (OA) is a prevalent degenerative joint disorder associated with pain, impaired proprioception, and reduced physical function. While closed kinetic chain exercises (CKCEs) are commonly prescribed to enhance joint stability, their weight-bearing nature may exacerbate symptoms. Graded weight-bearing exercises (GWBEs) using anti-gravity treadmill training provide a novel approach to reduce joint loading while maintaining functional mobility. This study aimed to evaluate the effectiveness of GWBEs compared with CKCEs and open kinetic chain exercises (OKCEs) on pain, function, proprioception, and quadricep strength in patients with knee OA. **Methods:** Forty-five adults aged 40–60 years with radiographically confirmed knee OA were randomized into three groups: (1) GWBE + OKCE, (2) CKCE + OKCE, or (3) OKCE alone. Interventions were conducted three times per week for six-weeks. Outcomes included pain (Visual Analogue Scale), physical function (Western Ontario and McMaster Universities Osteoarthritis Index, 6-Minute Walk Test), proprioception (joint repositioning error at 45°), and quadriceps strength (isokinetic peak torque at 60°, 120°, and 180°/s). **Results:** All groups demonstrated significant improvements in pain and function (*p* < 0.05). Proprioception improved in the GWBE + OKCE and CKCE + OKCE groups but not in the OKCE group. No significant changes were observed in quadriceps strength across groups. The GWBE + OKCE group showed significantly greater improvements in pain, function, and proprioception compared to both comparator groups (*p* < 0.05). **Conclusions:** GWBE combined with OKCE is more effective than CKCE + OKCE and OKCE alone in improving pain, function, and proprioception in patients with knee OA.

## 1. Introduction

Osteoarthritis (OA) is a progressive degenerative joint condition that affects the entire joint structure. It is characterized by the gradual deterioration of articular cartilage in synovial joints, accompanied by mild but persistent synovial inflammation, osteophyte formation, and subchondral bone sclerosis [1]. Knee osteoarthritis (knee OA) is the most common form of OA, primarily affecting the subchondral bone and articular cartilage of the knee joint [2]. Its prevalence is increasing globally, largely due to longer life expectancy and rising body mass index (BMI), which contribute to greater mechanical loading and joint degeneration [1]. Among adults over the age of 40, the global prevalence and incidence of knee OA are estimated at 22.9% and 22.3%, respectively [3]. In Saudi Arabia, the condition is highly prevalent, with reported rates of 60.9% in women and 53.3% in men, affecting individuals aged 30–90 years, with an average age of 49 years [4].

The progression of joint arthritis is primarily associated with mechanisms involving the detrimental effects of normal loading on compromised cartilage or abnormal loading on healthy cartilage [1]. The clinical signs and symptoms of knee OA include pain and functional impairment [2], joint irritation [5], joint degeneration [6], and joint instability [7]. These manifestations can significantly limit activities of daily living and may ultimately lead to functional decline and disability [2]. To address these disabling manifestations, treatment strategies for knee OA commonly include anti-inflammatory medications, therapeutic exercise programs, weight management, and electrotherapy modalities aimed at alleviating symptoms and preserving joint function.

Current evidence-based guidelines recommend various therapeutic exercise modalities for knee OA, such as closed kinetic chain exercises (CKCE), open kinetic chain exercises (OKCE), and aerobic and strengthening programs [8]. CKCE involve movements where the feet remain in contact with a fixed surface, generating compressive forces through the lower limb kinetic chain [9]. These exercises are beneficial for enhancing muscle strength, joint position sense, and neuromuscular control [9]. However, CKCE may be challenging for individuals with knee OA, as they can exacerbate pain, inflammation, and swelling due to increased joint loading [8,9]. Although widely implemented, a recent systematic review and meta-analysis indicated that the evidence supporting the effectiveness of CKCE on pain and function remains of low quality due to methodological limitations, heterogeneity, and limited data on proprioception [10]. To address these limitations, body weight-supported exercise modalities, such as the anti-gravity treadmill, have been proposed [8]. This technology permits adjustable body weight support (up to 80%), allowing patients to perform graded weight-bearing exercises (GWBE) while reducing joint load and pain [8]. GWBE offer both biomechanical and physiological advantages by mitigating mechanical stress on knee joints and preserving functional mobility. Evidence suggests that reducing joint load during exercise can alleviate pain and enhance physical function in individuals with knee OA [2,9]. Additionally, GWBE may improve joint circulation, promote anti-inflammatory effects [11], enhance muscle strength, and support proprioceptive function [8]. These exercises also activate neurophysiological pathways involved in pain modulation [12].

Despite growing interest in GWBE, there is a clear gap in the literature concerning direct comparisons between GWBE and traditional exercise modalities (CKCE and OKCE) across various clinical outcomes in knee OA populations. Specifically, no studies have systematically evaluated the efficacy of GWBE as a modified form of CKCE for improving pain, function, proprioception, and muscle strength in this patient group. Therefore, the aim of this study is to evaluate the effectiveness of GWBE compared to CKCE and OKCE in the management of knee OA. By examining pain, functional capacity, proprioception, and muscle strength, this trial seeks to provide evidence-based insights that may inform clinical decision-making and enhance therapeutic strategies for knee OA.

## 2. Materials and Methods

### 2.1. Study Design

This study was designed as a single-blinded, randomized controlled trial (RCT). The full trial protocol is registered with the Clinical Trials Registry under registration number NCT05671146. The study adhered to the CONSORT guidelines and was conducted in accordance with the principles outlined in the Declaration of Helsinki. Ethical approval was obtained from the Institutional Review Board of Umm Al-Qura University (approval number: HAPO-02-K-012-2023-06-1690). Written informed consent was obtained from all participants prior to enrolment.

### 2.2. Recruitment, Allocation, and Blinding

Patients diagnosed with knee OA were referred by independent physicians from the Orthopedic Department at Umm Al-Qura University Medical Clinic. Eligibility screening was conducted by an independent physician, who also oversaw participant enrolment and group allocation. Following completion of baseline assessments, participants were randomly assigned to one of three intervention groups: Group I, which received GWBE combined with OKCE (GWBE + OKCE group); Group II, which received CKCE combined with OKCE (CKCE + OKCE group); and Group III, which received only OKCE (OKCE group).

Randomization was performed using a computer-generated block randomization method with a block size of six to ensure balanced group sizes throughout the enrolment period. The random allocation sequence was generated by an independent statistician who had no involvement in the study’s implementation or data analysis. Allocation concealment was maintained using sealed, opaque, sequentially numbered envelopes, which were opened only after participants completed all baseline assessments. To minimize bias, the outcome assessors were blinded to group allocation. A single physiotherapist, who was not involved in outcome assessments, delivered all intervention sessions. All outcome measures were evaluated by the same trained assessors throughout the study. Two physiotherapists served as assessors for all outcome measurements. Prior to data collection, both underwent standardized training sessions to ensure full understanding of the study protocol, measurement procedures, and use of equipment. They adhered to a predefined protocol established before the study began and used the same materials and standardized assessment environment. Depending on their availability and work schedules, either assessor performed the evaluations. This approach ensured procedural consistency and minimized inter-assessor variability or potential measurement bias throughout the study.

### 2.3. Power Calculation

The sample size was estimated using G*Power version 3.1 for Windows. Based on an effect size of 0.25, an alpha error probability of 0.05, and a statistical power of 90%, the required sample size to detect changes in pain (the primary outcome) across three independent intervention groups affecting five dependent variables was calculated to be 36 participants [13]. To account for potential dropouts, a 15% dropout rate (based on rates observed in similar mid-term studies) was anticipated, and the initial sample size was adjusted accordingly to ensure adequate statistical power.

### 2.4. Eligibility Criteria

Participants were eligible for inclusion in the study if they met the following criteria: (1) experienced chronic unilateral or bilateral knee pain for at least six months; (2) had a clinical diagnosis of knee OA, irrespective of gender, race, educational level, or socioeconomic status; (3) exhibited radiographic evidence of grade II or III knee OA according to the Kellgren and Lawrence grading system; (4) were between 40 and 60 years of age; and (5) were willing to actively participate in the study and discontinue any medications at least one week prior to participation.

Exclusion criteria included: (1) the presence of any significant medical conditions (such as hypertension, asthma, cardiovascular disease, or systemic illness) that could pose a risk during physical testing; (2) history of knee surgery or intra-articular injection within the past six months; (3) previous knee fracture or malignancy; (4) inability to ambulate without assistance; and (5) inability to commit to the study schedule and protocol.

### 2.5. Interventions

The intervention protocols were developed in accordance with recent clinical exercise guidelines for individuals with knee OA, which recommend weight-bearing activities, progressive resistance training targeting major lower limb muscle groups, and aerobic exercises [1,14]. The specific exercise regimen varied according to the assigned treatment group.

**Figure 1:**
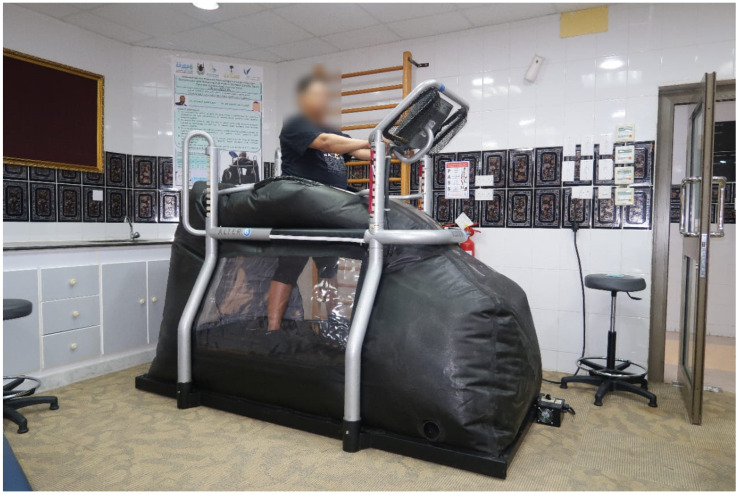
Anti-gravity treadmill used in the GWBE + OKCE group.

Group I: Participants in this group received a combination of GWBE and OKCE (GWBE + OKCE group). The GWBE was performed using an anti-gravity treadmill (Alter-G Pro 200, Alter-G Inc., Fremont, CA, USA) (Figure 1). Each session began with a 5 min warm-up (speed: 0–2 mph), followed by 15 min of walking (speed: 2.0 mph), and ended with a 5 min cooldown (speed: 2–0 mph) [8]. The level of body weight support was initially set within each participant’s pain-free tolerance and was gradually reduced by 10% per week over the six-week period. Each session concluded with 10 min of stretching and cool-down activities. Participants were encouraged to report any adverse symptoms, such as muscle soreness, joint tenderness, or increased pain. The OKCE component administered to this group is described in detail under Group III (OKCE group) below.

Group II: Participants in this group received CKCE, combined with OKCE (CKCE + OKCE group). The CKCE protocol included three primary exercises performed in standing or supine positions: mini-squats, wall pushes, and single-leg standing. For the mini-squat, participants stood in front of a fixed support and flexed both knees to approximately 30°, holding the position for 10 counts before relaxing, repeated 10 times [15]. In the wall push exercise, participants lay supine with hips and knees flexed to 90°, pushing against a wall for 10 counts before relaxing, also repeated 10 times [15]. For the single-leg stance, participants stood beside a stable support, lifted one leg, and held the position for 10 counts, repeated 10 times [15]. Most exercises were performed in weight-bearing positions, with or without external support. Similarly to Group I, the OKCE component provided to this group is described in detail under Group III (OKCE group) below.

Group III: This group received only OKCE, which included both stretching and strengthening activities targeting the knee flexors and extensors (OKCE group). The OKCE program comprised four exercises performed in sitting or supine positions: knee extensions, straight leg raises [13], isometric quadriceps strengthening [13], and hamstring stretching [15]. All exercises were performed in non-weight-bearing positions, with each exercise conducted in three sets of 10 repetitions.

An experienced physiotherapist supervised each session, ensuring correct exercise technique, appropriate dosage, and individualized progression. Additional precautions were taken for participants with limited mobility, restricted weight-bearing capacity, or a higher risk of falls, including the provision of support devices when necessary. All participants received an illustrated exercise booklet on the first day, detailing the prescribed exercises. Compliance was monitored using individual exercise logs and physiotherapist reports. Participants were instructed to report any adverse events, such as discomfort, joint pain, falls, or other injuries occurring during or outside the exercise sessions. All treatment sessions for the three groups were conducted three times per week over a six-week period. Each session lasted between 20 and 30 min, with 30 s rest intervals between exercises. Participants who missed three consecutive sessions without valid justification were excluded from the study.

### 2.6. Outcome Measures

In participants with bilateral knee OA, the more symptomatic knee was identified based on self-reported pain and clinical examination. This knee was selected for all outcome measurements and intervention to maintain consistency and comparability among participants. The following were the outcome measures used in this study.

Pain: Pain intensity was assessed using the Visual Analogue Scale (VAS), represented by a 10 cm horizontal line. The scale ranged from 0 (indicating no pain) to 10 (indicating the most severe pain imaginable). Participants were instructed to mark the point on the line that best represented their current level of knee pain. The VAS has been shown to be a valid and reliable tool for assessing pain in individuals with chronic musculoskeletal conditions [16].

Knee Function: Knee function was evaluated using two validated methods. The Western Ontario and McMaster Universities Arthritis Index (WOMAC) was used to assess self-reported pain, stiffness, and physical function. The WOMAC includes three subscales: five items for pain, two for stiffness, and 17 for physical function. Each item is rated on a five-point Likert scale (None, Mild, Moderate, Severe, Extreme), with higher scores indicating greater disability and poorer quality of life [17]. The Six-Minute Walk Test (6-MWT) was employed to measure functional walking capacity. This test is widely recognized as a valid and reliable indicator of physical function in patients with knee OA and is endorsed by the American College of Rheumatology [18,19]. The test was performed in a controlled laboratory setting on a flat surface with a marked 10 m distance. Participants were instructed to walk as far as possible in six minutes without receiving any encouragement or time feedback. The total distance walked was recorded [20].

Proprioception: Knee joint proprioception was assessed using the isokinetic dynamometer (Biodex system 4), which is considered a reliable instrument for measuring joint position sense [21]. During testing, participants were seated and secured using straps across the trunk, chest, pelvis, and hips to prevent movement. The dynamometer’s lever arm was attached to the lower leg at the level of the malleoli, and its axis aligned with the lateral epicondyle of the femur. The seatback was inclined at an angle between 70° and 85°, and the device was set to a fixed orientation of 90°. Proprioceptive accuracy was measured at a target knee flexion angle of 45° [22]. Participants first performed three practice trials. Once familiarized, they were blindfolded and instructed to extend their knee from 90° flexion (defined as full flexion) to the target angle. The angular error was calculated as the absolute difference between the target and the reproduced angle (position–reposition error), for both dominant and non-dominant limbs.

Muscle Strength: Quadriceps muscle strength was assessed using the isokinetic dynamometer (Biodex system 4), which provides accurate measurements of muscle torque (MT) [21]. Participants were seated with a 15° backward tilt and secured at the upper chest, pelvis, and distal femur. After a 5 min warm-up involving active, unloaded knee flexion and extension, participants performed maximal concentric contractions of the quadriceps across a specified range of motion. Peak torque was measured at angular velocities of 60°/s, 120°/s, and 180°/s, using three repetitions per speed. The highest value at each velocity was recorded for analysis [23,24].

### 2.7. Statistical Analysis

Descriptive statistics, including age, height, weight, BMI, and all outcome variables (VAS, WOMAC, 6-MWT, proprioception, and quadriceps muscle torque), were analyzed using IBM SPSS Statistics for Windows (version 26.0). Normality of the data distribution was assessed prior to further analysis. All outcome measurements were recorded at baseline (pre-intervention) and after six weeks of intervention (post-intervention). To compare outcomes across the three intervention groups and over time, a multivariate analysis of variance (MANOVA) was conducted. Pairwise comparisons of group means were performed, and post hoc analyses using the Bonferroni correction were applied to identify significant differences between groups. Results were reported as mean ± standard deviation (SD), and a *p*-value ≤ 0.05 was considered statistically significant.

## 3. Results

**Figure 2:**
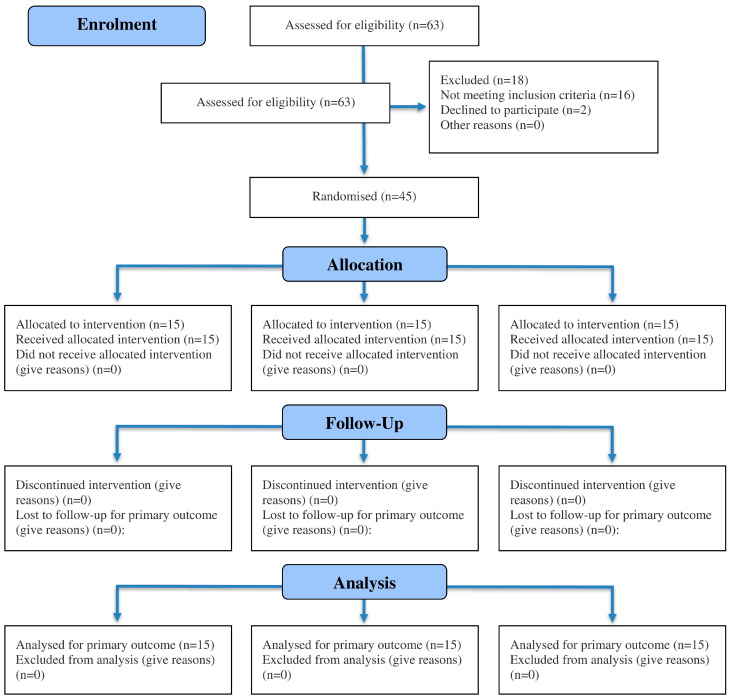
CONSORT flow diagram of participant recruitment, randomization, allocation, and follow-up.

The baseline demographic characteristics of the participants are presented in Table 1. The participant recruitment, randomization, allocation, and follow-up processes are detailed in the CONSORT flow chart (Figure 2). Homogeneity of variance was assessed using Levene’s Test, which revealed no significant differences across the groups. The Shapiro–Wilk test indicated a non-significant result (*p* > 0.05), confirming that the data were normally distributed.

All measured variables (VAS, WOMAC, 6-MWT, proprioception, and quadriceps muscle torque) showed no statistically significant differences between groups at baseline (*p* > 0.05). This absence of pre-treatment differences is critical, as it confirms the initial comparability of the groups and supports the attribution of any post-treatment changes to the interventions themselves rather than to pre-existing differences.

At post-intervention, all groups demonstrated significant improvements in several outcome measures (Table 2). Pain levels, as measured by the VAS, significantly decreased in the GWBE + OKCE group (*p* = 0.001; mean = 1.687 ± 0.42), the CKCE + OKCE group (*p* = 0.0001; mean = 2.2 ± 0.66), and the OKCE group (*p* = 0.0001; mean = 2.36 ± 0.39). Functional ability, assessed using the WOMAC scale, also improved significantly in the GWBE + OKCE group (*p* = 0.0001; mean = 37.73 ± 4.6), the CKCE + OKCE group (*p* = 0.0001; mean = 42.73 ± 3.26), and the OKCE group (*p* = 0.0001; mean = 45.7 ± 3.73). Similarly, functional performance assessed by the 6-MWT showed significant improvement in all three groups: GWBE + OKCE (*p* = 0.0001; mean = 337.6 ± 11.8), CKCE + OKCE (*p* = 0.0001; mean = 321 ± 14.4), and OKCE (*p* = 0.0001; mean = 314 ± 14.64).

Regarding proprioception (measured as joint repositioning error), significant improvements were observed in both the GWBE + OKCE group (*p* = 0.0001; mean = 4.67 ± 0.35) and the CKCE + OKCE group (*p* = 0.0001; mean = 5.92 ± 0.23). In contrast, the OKCE group demonstrated a non-significant improvement in proprioception (*p* = 0.08). Post-intervention measurements of quadriceps muscle strength at angular velocities of 60°/s, 120°/s, and 180°/s revealed no statistically significant differences among the three groups (*p* > 0.05). This lack of significance suggests that the various interventions had a comparable effect on muscle strength development across groups.

The post hoc Bonferroni analysis further revealed significant between-group differences in several outcome measures (Table 3). Pain levels were significantly lower in the GWBE + OKCE group compared to both the CKCE + OKCE group (*p* = 0.02) and the OKCE group (*p* = 0.002). WOMAC scores also demonstrated significant improvement in the GWBE + OKCE group when compared to both the CKCE + OKCE group (*p* = 0.002) and the OKCE group (*p* = 0.001). For the 6-MWT, the GWBE + OKCE group showed significantly greater improvements than both the CKCE + OKCE group (*p* = 0.005) and the OKCE group (*p* = 0.001). Additionally, the GWBE + OKCE group exhibited significantly greater improvements in proprioception compared to the CKCE + OKCE group (*p* = 0.001).

These findings indicate that the GWBE + OKCE intervention was more effective in improving pain, function, walking capacity, and proprioception compared to both the CKCE + OKCE and OKCE-alone interventions. The absence of significant differences in pain and function between the CKCE + OKCE and OKCE groups suggests that these two modalities yielded comparable outcomes in these domains. Overall, these results underscore the differential efficacy of exercise modalities in managing knee OA and provide valuable insights for optimizing therapeutic interventions.

## 4. Discussion

This study aimed to investigate the effects of GWBE program on pain, function, proprioception, and muscle strength in patients with knee OA. Based on the existing literature, this is the first RCT to directly compare the impact of GWBE combined with OKCE against other established exercise modalities (CKCE + OKCE and OKCE alone) across multiple clinically relevant outcomes. All three intervention groups (GWBE + OKCE, CKCE + OKCE, and OKCE) demonstrated significant reductions in pain (VAS), and improvements in functional performance (WOMAC and 6-MWT). Notably, joint proprioception improved significantly in the GWBE + OKCE and CKCE + OKCE groups, whereas no significant proprioceptive gains were observed in the OKCE group. Among all groups, GWBE + OKCE produced the most pronounced improvements across four of the five assessed outcome measures. These findings suggest a superior efficacy of GWBE in the comprehensive management of knee OA symptoms.

The improvements observed in pain and function are consistent with earlier findings by Bennell et al. [10], Lai et al. [25], and Moreira et al. [26], which emphasize the efficacy of tailored exercise programs in reducing symptoms and enhancing physical performance in knee OA patients. The superior outcomes observed in the GWBE + OKCE group may be attributed to the use of antigravity treadmill training, which reduces joint load while maintaining functional, weight-bearing movement. This approach leverages body-weight support to modulate gravitational forces, thereby minimizing joint stress and facilitating pain-free participation in exercise. By preserving natural gait patterns, GWBE promotes biomechanical efficiency and supports improvements in functional endurance. These adaptations are reflected in the significant gains observed in the 6-MWT, a validated and reliable measure of aerobic capacity and overall functional status in individuals with knee OA [8,12,19,20].

A particularly notable finding was the significant improvement in proprioception in both the GWBE + OKCE and CKCE + OKCE groups, but not in the OKCE group. This aligns with earlier studies that suggest CKCE and weight-bearing tasks promote joint stability through enhanced proprioceptive feedback [22,23]. The failure of the OKCE group to achieve similar gains in proprioception contrasts with findings from Desai et al. [27] and highlights the limitations of non-weight-bearing exercises in stimulating sensory feedback mechanisms essential for joint control. The physiological benefits of GWBE may stem from neuromuscular adaptations under altered gravitational loading. The modulation of spinal reflex activity—particularly the H-reflex and F-wave—has been implicated in improved sensorimotor integration during body-weight-supported exercises [28]. These mechanisms likely contribute to the enhanced proprioceptive accuracy and motor control observed in the GWBE + OKCE group. Moreover, the improvements in proprioception in both the GWBE + OKCE and CKCE + OKCE groups align with findings by Ferreira et al. [29] and Molla et al. [30], who emphasized the critical role of proprioceptive training in enhancing balance, joint stability, and mobility among individuals with knee OA. Our findings further support and expand upon a recent systematic review and meta-analysis, which concluded that while CKCE may offer some benefits for proprioception and function, the overall evidence base remains low in quality and limited in scope [10]. These findings indicate that proprioceptive enhancement may depend on the degree of weight-bearing and sensorimotor engagement during exercise. Further research should aim to isolate and compare the independent effects of GWBE, CKCE, and OKCE to better understand their respective roles in improving proprioception in knee OA.

Interestingly, muscle torque did not significantly improve in any group, which contrasts with previous studies by Jan et al. [31,32] and Lin et al. [33] that reported significant strength gains following CKCE protocols in patients with knee OA. This discrepancy may be attributed to several factors, including differences in program duration, exercise intensity, and participant characteristics such as baseline muscle strength and physical conditioning. The absence of significant improvements in torque observed in the present study suggests that longer-duration or more targeted resistance training protocols may be required to elicit measurable gains in muscle strength. These findings underscore the need for future research to explore optimal dosing, progression, and individualization of exercise interventions to maximize strength outcomes in this population.

The GWBE program represents an innovative approach to knee OA rehabilitation by combining the functional benefits of traditional closed kinetic chain exercises CKCE with the supportive unloading properties of hydrotherapy. As demonstrated in studies by Patil et al. [34] and Miyoshi et al. [35], this integrated approach can effectively reduce joint loading while preserving natural movement patterns. Furthermore, evidence from other clinical populations—including patients with ankle and tibial plateau fractures [36], individuals with Parkinson’s disease [37], and stroke survivors [38]—has shown that anti-gravity treadmill training can improve gait performance, mitigate muscle atrophy, and enhance cardiorespiratory fitness. These findings reinforce the broader utility of antigravity-supported rehabilitation and suggest that GWBE may offer a comprehensive and effective therapeutic strategy for improving physical function in individuals with knee OA.

Collectively, the present findings contribute new insights into the application of GWBE + OKCE, CKCE + OKCE, and OKCE in patients with knee OA. These results support the effectiveness of these exercise modalities in reducing pain and improving function, with additional benefits in proprioception for GWBE + OKCE and CKCE + OKCE. However, the limited effects on muscle torque warrant careful reconsideration of exercise design. The outcomes highlight the importance of adopting multifaceted, evidence-based strategies to optimize rehabilitation and minimize functional limitations in this population.

This study supports the use of GWBE + OKCE, CKCE + OKCE, and OKCE as effective exercise modalities for reducing pain and improving function in patients with knee OA. GWBE + OKCE and CKCE + OKCE were particularly effective in enhancing proprioception, with GWBE + OKCE yielding superior results in four of five clinical outcome measures. Given its pain-free nature and capacity to preserve gait mechanics, GWBE may serve as a valuable alternative to traditional CKCE. Physical therapists may consider combining GWBE with OKCE to achieve optimal rehabilitation outcomes in terms of pain relief, functional enhancement, proprioceptive recovery, and prevention of disability progression.

This study has several limitations that should be acknowledged. First, the duration of the intervention may have been insufficient to capture long-term adaptations, particularly in muscle strength outcomes. Second, the sample exhibited a gender imbalance, with a predominance of female participants, which may limit the generalizability of the findings to the broader knee OA population. Third, participants’ perceptions of the anti-gravity treadmill experience (e.g., the sensation of lower body pressure and overall comfort) were not assessed, despite their potential influence on adherence and engagement with the intervention. Fourth, the use of home-based heat therapy for pain relief was not monitored or standardized, which may have influenced participants’ pain levels during outcome assessments. Fifth, this study did not include two additional comparison groups: one combining GWBE and CKCE, and another including only CKCE. Future studies with a larger sample size should consider including these groups to provide a more comprehensive comparison and to better understand the combined or independent effects of these exercise types on pain, function, and proprioception in individuals with knee OA. Sixth, the study did not include an isokinetic exercise training group, although muscle strength was assessed using an isokinetic dynamometer. All interventions involved mainly isotonic contractions. Future research should include an isokinetic training group to determine whether this modality offers additional advantages in improving quadriceps strength and overall function in knee OA. Lastly, the sample size in this study was determined a priori using a power analysis to achieve adequate statistical power for detecting changes in the primary outcome. However, the relatively small number of participants in each group may restrict the generalizability of the findings to broader clinical populations. Future studies with larger and more diverse samples are recommended to confirm these results and enhance external validity.

## 5. Conclusions

This RCT demonstrated that all three exercise interventions (GWBE + OKCE, CKCE + OKCE, and OKCE alone) were effective in reducing pain and improving functional performance in patients with knee OA. Notably, only the GWBE + OKCE and CKCE + OKCE groups showed significant improvements in proprioception, highlighting the added value of weight-bearing components in enhancing sensorimotor outcomes. Among the interventions, GWBE + OKCE produced the most consistent and superior improvements across multiple clinical outcomes, including pain, function, walking capacity, and proprioception, while none of the exercise interventions yielded significant gains in quadriceps muscle torque. These findings suggest that GWBE, delivered via an anti-gravity treadmill and combined with OKCE, offers a promising and well-tolerated alternative to conventional rehabilitation approaches. By enabling pain-free, gait-specific weight-bearing activity, GWBE may help overcome common barriers to exercise adherence in patients with knee OA. Future research should evaluate the long-term effects of GWBE, explore optimal training parameters, and assess patient-reported outcomes such as comfort, satisfaction, and adherence to inform broader clinical implementation.

## Figures and Tables

**Table 1:** The baseline demographic characteristics of the participants.

Characteristics	GWBE + OCKE	CKCE + OCKE	OCKE	*p* Value
Age	52.6 ± 5.7	51.4 ± 4.9	51.8 ± 6.3	0.840
Female, N (%)	14 (93%)	10 (66%)	12 (85%)	-
Weight	89.4 ± 18.6	97.2 ± 13.8	93.4 ± 17.3	0.447
Height	165.6 ± 9.3	167 ± 9.8	165.4 ± 8.7	0.876
BMI	37.6 ± 5.8	37.8 ± 6.3	37.3 ± 5.9	0.974
Duration of illness	38.3 ± 8.7	42.7 ± 9.2	41.2 ± 7.6	0.365
K-L score—grade II	9 (60%)	11 (73%)	8 (53%)	-
K-L score—grade III	6 (40%)	4 (40%)	7 (47%)	-

GWBE: graded weight-bearing exercises; OKCE: open kinetic chain exercises; CKCE: closed kinetic chain exercises; BMI: body mass index.

**Table 2:** Within-group pre- and post-treatment changes in outcome measures among treatment groups.

Variable		GWBE + OCKE	CKCE + OCKE	OCKE	*p* Value
VAS	Pre-treatment	5.94 ± 0.71	6.04 ± 0.71	6.27 ± 0.99	0.5274
	Post-treatment	1.69 ± 0.42	2.20 ± 0.66	2.36 ± 0.39	0.0021 ^a^
	Mean difference	4.253	3.847	3.913	
	*p* value	<0.0001 ^b^	<0.0001 ^b^	<0.0001 ^b^	
WOMAC	Pre-treatment	61.13 ± 4.32	62.00 ± 3.87	61.86 ± 3.50	0.8088
	Post-treatment	37.73 ± 4.61	42.73 ± 3.26	45.73 ± 3.73	<0.0001 ^a^
	Mean difference	23.4	19	16.133	
	*p* value	<0.0001 ^b^	<0.0001 ^b^	<0.0001 ^b^	
6-MWT	Pre-treatment	306.40 ± 18.38	303.53 ± 16.04	305.30 ± 13.25	0.8857
	Post-treatment	337.67 ± 11.84	321.00 ± 14.45	314.00 ± 14.64	<0.0001 ^a^
	Mean difference	−31.267	−17.467	−8.733	
	*p* value	<0.0001 ^b^	<0.0001 ^b^	<0.0001 ^b^	
Prop-45	Pre-treatment	6.10 ± 0.18	6.22 ± 0.21	6.24 ± 0.23	0.1304
	Post-treatment	4.67 ± 0.36	5.93 ± 0.23	6.17 ± 0.28	<0.0001 ^a^
	Mean difference	1.427	0.2933	0.07333	
	*p* value	<0.0001 ^b^	<0.0001 ^b^	0.0853	
MT-60°/s	Pre-treatment	78.87 ± 7.77	76.33 ± 7.02	73.80 ± 6.37	0.1592
	Post-treatment	80.06 ± 8.77	76.73 ± 6.96	73.86 ± 6.61	0.0893
	Mean difference	−1.2	−0.4	−0.066	
	*p* value	0.0510	0.4793	0.8800	
MT-120°/s	Pre-treatment	59.46 ± 4.29	60.80 ± 5.07	61.60 ± 5.70	0.5110
	Post-treatment	60.06 ± 4.89	61.93 ± 5.80	61.46 ± 6.18	0.6450
	Mean difference	−0.6	−1.13	0.133	
	*p* value	0.1442	0.2254	0.7284	
MT-180°/s	Pre-treatment	45.73 ± 5.23	46.60 ± 3.66	44.33 ± 5.39	0.4377
	Post-treatment	45.86 ± 5.43	47.66 ± 3.86	44.60 ± 5.95	0.2735
	Mean difference	−0.1333	−1.06	−0.2667	
	*p* value	0.8178	0.4400	0.6330	

GWBE: graded weight-bearing exercises; OKCE: open kinetic chain exercises; CKCE: closed kinetic chain exercises; VAS: visual analogue scale; WOMAC: Western Ontario and McMaster universities osteoarthritis; 6-MWT: 6 min walk test; Prop-45: proprioception at 45°; MT: muscle torque. ^a^ Significant difference among treatment intervals (multivariate tests; *p* ≤ 0.05). ^b^ Significant difference between two treatment groups (paired *t* test; *p* ≤ 0.05).

**Table 3:** Between-group pairwise comparisons of pre- and post-treatment outcome measures among treatment groups.

Variable		Group	Comparison	MD	*p* Value
VAS	Pre-treatment	GWBE + OCKE	CKCE + OCKE	−0.106	*p* > 0.05 ^b^
		GWBE + OCKE	OCKE	−0.33	*p* > 0.05 ^b^
		CKCE + OCKE	OCKE	−0.22	*p* > 0.05 ^b^
	Post-treatment	GWBE + OCKE	CKCE + OCKE	−0.5133	0.0250 ^a^
		GWBE + OCKE	OCKE	−0.6733	0.0020 ^a^
		CKCE + OCKE	OCKE	−0.16	*p* > 0.05 ^b^
WOMAC	Pre-treatment	GWBE + OCKE	CKCE + OCKE	−0.8667	*p* > 0.05 ^b^
		GWBE + OCKE	OCKE	−0.7333	*p* > 0.05 ^b^
		CKCE + OCKE	OCKE	0.1333	*p* > 0.05 ^b^
	Post-treatment	GWBE + OCKE	CKCE + OCKE	−5.267	0.0020 ^a^
		GWBE + OCKE	OCKE	−8	0.0001 ^a^
		CKCE + OCKE	OCKE	−2.73	*p* > 0.05 ^b^
6-MWT	Pre-treatment	GWBE + OCKE	CKCE + OCKE	2.867	*p* > 0.05 ^b^
		GWBE + OCKE	OCKE	1.133	*p* > 0.05 ^b^
		CKCE + OCKE	OCKE	−1.733	*p* > 0.05 ^b^
	Post-treatment	GWBE + OCKE	CKCE + OCKE	16.667	0.0050 ^a^
		GWBE + OCKE	OCKE	23.667	0.0001 ^a^
		CKCE + OCKE	OCKE	7	*p* > 0.05 ^b^
Prop-45	Pre-treatment	GWBE + OCKE	CKCE + OCKE	−0.1193	*p* > 0.05 ^b^
		GWBE + OCKE	OCKE	−0.146	*p* > 0.05 ^b^
		CKCE + OCKE	OCKE	−0.02667	*p* > 0.05 ^b^
	Post-treatment	GWBE + OCKE	CKCE + OCKE	−1.253	0.0001 ^a^
		GWBE + OCKE	OCKE	−1.5	0.0001 ^a^
		CKCE + OCKE	OCKE	−0.2467	*p* > 0.05 ^b^
MT-60°/s	Pre-treatment	GWBE + OCKE	CKCE + OCKE	2.533	*p* > 0.05 ^b^
		GWBE + OCKE	OCKE	5.067	*p* > 0.05 ^b^
		CKCE + OCKE	OCKE	2.533	*p* > 0.05 ^b^
	Post-treatment	GWBE + OCKE	CKCE + OCKE	3.333	*p* > 0.05 ^b^
		GWBE + OCKE	OCKE	6.2	*p* > 0.05 ^b^
		CKCE + OCKE	OCKE	2.86	*p* > 0.05 ^b^
MT-120°/s	Pre-treatment	GWBE + OCKE	CKCE + OCKE	−1.33	*p* > 0.05 ^b^
		GWBE + OCKE	OCKE	−2.13	*p* > 0.05 ^b^
		CKCE + OCKE	OCKE	−0.8	*p* > 0.05 ^b^
	Post-treatment	GWBE + OCKE	CKCE + OCKE	−1.86	*p* > 0.05 ^b^
		GWBE + OCKE	OCKE	−1.4	*p* > 0.05 ^b^
		CKCE + OCKE	OCKE	0.46	*p* > 0.05 ^b^
MT-180°/s	Pre-treatment	GWBE + OCKE	CKCE + OCKE	−0.86	*p* > 0.05 ^b^
		GWBE + OCKE	OCKE	1.4	*p* > 0.05 ^b^
		CKCE + OCKE	OCKE	2.2	*p* > 0.05 ^b^
	Post-treatment	GWBE + OCKE	CKCE + OCKE	−1.8	*p* > 0.05 ^b^
		GWBE + OCKE	OCKE	1.26	*p* > 0.05 ^b^
		CKCE + OCKE	OCKE	3.06	*p* > 0.05 ^b^

MD: mean difference; VAS: visual analogue scale; WOMAC: Western Ontario and McMaster universities osteoarthritis; 6-MWT: 6 min walk test; Prop-45: proprioception at 45°; MT: muscle torque; GWBE: graded weight-bearing exercises; OKCE: open kinetic chain exercises; CKCE: closed kinetic chain exercises. ^a^ Significant difference among treatment intervals (post hoc Bonferroni test; *p* ≤ 0.05). ^b^ Non-significant difference.

## Data Availability

Data of this study is secure with the first author and available on request.

## Abbreviations

The following abbreviations are used in this manuscript:

OA	Osteoarthritis
Knee OA	Knee osteoarthritis
BMI	body mass index
CKCE	Closed kinetic chain exercises
OKCE	Open kinetic chain exercises
GWBE	Graded weight-bearing exercises
RCT	Randomized controlled trial
VAS	Visual Analogue Scale
WOMAC	Western Ontario and McMaster Universities Arthritis Index
6-MWT	Six-Minute Walk Test
MT	Muscle torque
MANOVA	Multivariate analysis of variance
SD	Standard deviation
Prop-45	Proprioception at 45°
